# In search of environmental risk factors for obsessive-compulsive disorder: study protocol for the OCDTWIN project

**DOI:** 10.1186/s12888-023-04897-4

**Published:** 2023-06-16

**Authors:** David Mataix-Cols, Lorena Fernández de la Cruz, Elles De Schipper, Ralf Kuja-Halkola, Cynthia M. Bulik, James J. Crowley, Janina Neufeld, Christian Rück, Kristiina Tammimies, Paul Lichtenstein, Sven Bölte, Jan C. Beucke

**Affiliations:** 1 Department of Clinical Neuroscience, Centre for Psychiatry Research, Karolinska Institutet, Stockholm, Sweden; 2 Stockholm Health Care Services, Region Stockholm, Stockholm, Sweden; 3 Department of Clinical Sciences, Lund University, Lund, Sweden; 4 Department of Medical Epidemiology and Biostatistics, Karolinska Institutet, Stockholm, Sweden; 5 Department of Psychiatry, University of North Carolina at Chapel Hill, Chapel Hill, NC USA; 6 Department of Nutrition, University of North Carolina at Chapel Hill, Chapel Hill, NC USA; 7 Department of Genetics, University of North Carolina at Chapel Hill, Chapel Hill, USA; 8 Center of Neurodevelopmental Disorders at Karolinska Institutet (KIND), Centre for Psychiatry Research, Department of Women’s and Children’s Health, Karolinska Institutet, Stockholm, Sweden; 9 Swedish Collegium for Advanced Study (SCAS), Uppsala, Sweden; 10 Astrid Lindgren Children’s Hospital, Karolinska University Hospital, Region Stockholm, Solna, Sweden; 11 Curtin Autism Research Group, Curtin School of Allied Health, Curtin University, Perth, WA Australia; 12 Department of Psychology, Humboldt-Universität zu Berlin, Berlin, Germany; 13 Institute for Systems Medicine, Faculty of Human Medicine, MSH Medical School Hamburg, Hamburg, Germany

**Keywords:** Obsessive-compulsive disorder, Monozygotic twins, Discordant twins, Environmental risk factors, Prevention, Early intervention

## Abstract

**Background:**

The causes of obsessive-compulsive disorder (OCD) remain unknown. Gene-searching efforts are well underway, but the identification of environmental risk factors is at least as important and should be a priority because some of them may be amenable to prevention or early intervention strategies. Genetically informative studies, particularly those employing the discordant monozygotic (MZ) twin design, are ideally suited to study environmental risk factors. This protocol paper describes the study rationale, aims, and methods of OCDTWIN, an open cohort of MZ twin pairs who are discordant for the diagnosis of OCD.

**Methods:**

OCDTWIN has two broad aims. In Aim 1, we are recruiting MZ twin pairs from across Sweden, conducting thorough clinical assessments, and building a biobank of biological specimens, including blood, saliva, urine, stool, hair, nails, and multimodal brain imaging. A wealth of early life exposures (e.g., perinatal variables, health-related information, psychosocial stressors) are available through linkage with the nationwide registers and the Swedish Twin Registry. Blood spots stored in the Swedish phenylketonuria (PKU) biobank will be available to extract DNA, proteins, and metabolites, providing an invaluable source of biomaterial taken at birth. In Aim 2, we will perform within-pair comparisons of discordant MZ twins, which will allow us to isolate unique environmental risk factors that are in the causal pathway to OCD, while strictly controlling for genetic and early shared environmental influences. To date (May 2023), 43 pairs of twins (21 discordant for OCD) have been recruited.

**Discussion:**

OCDTWIN hopes to generate unique insights into environmental risk factors that are in the causal pathway to OCD, some of which have the potential of being actionable targets.

## Background

Despite dedicated research and some breakthroughs in the scientific understanding of relevant neurobiological and psychosocial factors, the causes of obsessive-compulsive disorder (OCD) remain largely unknown. Efforts to identify associated genetic variants are well underway through unprecedented international collaboration [1–3]. The identification of specific environmental factors that confer risk, and may interact with genetic factors, is at least as important as identifying genetic variants [4]. This could be regarded as a priority because some environmental risk factors may be amenable to prevention or early intervention strategies [5]. Unfortunately, little progress has been made in this area, primarily because the identification of environmental risk factors that operate independently from genetic factors is challenging.

Genetically informative studies, specifically those employing the discordant monozygotic (MZ) twin design, are ideally suited to test whether the association between an environmental measure and an observed phenotype (e.g., OCD) is likely to be consistent with a causal effect since they provide strict control of both genetic and early shared environmental effects. In Sweden, we have begun an ambitious project to create the world’s first cohort of MZ twin pairs who are discordant for a diagnosis of OCD. In this protocol paper, we describe the study rationale, aims, and methods of the OCDTWIN project.

**Fig. 1:**
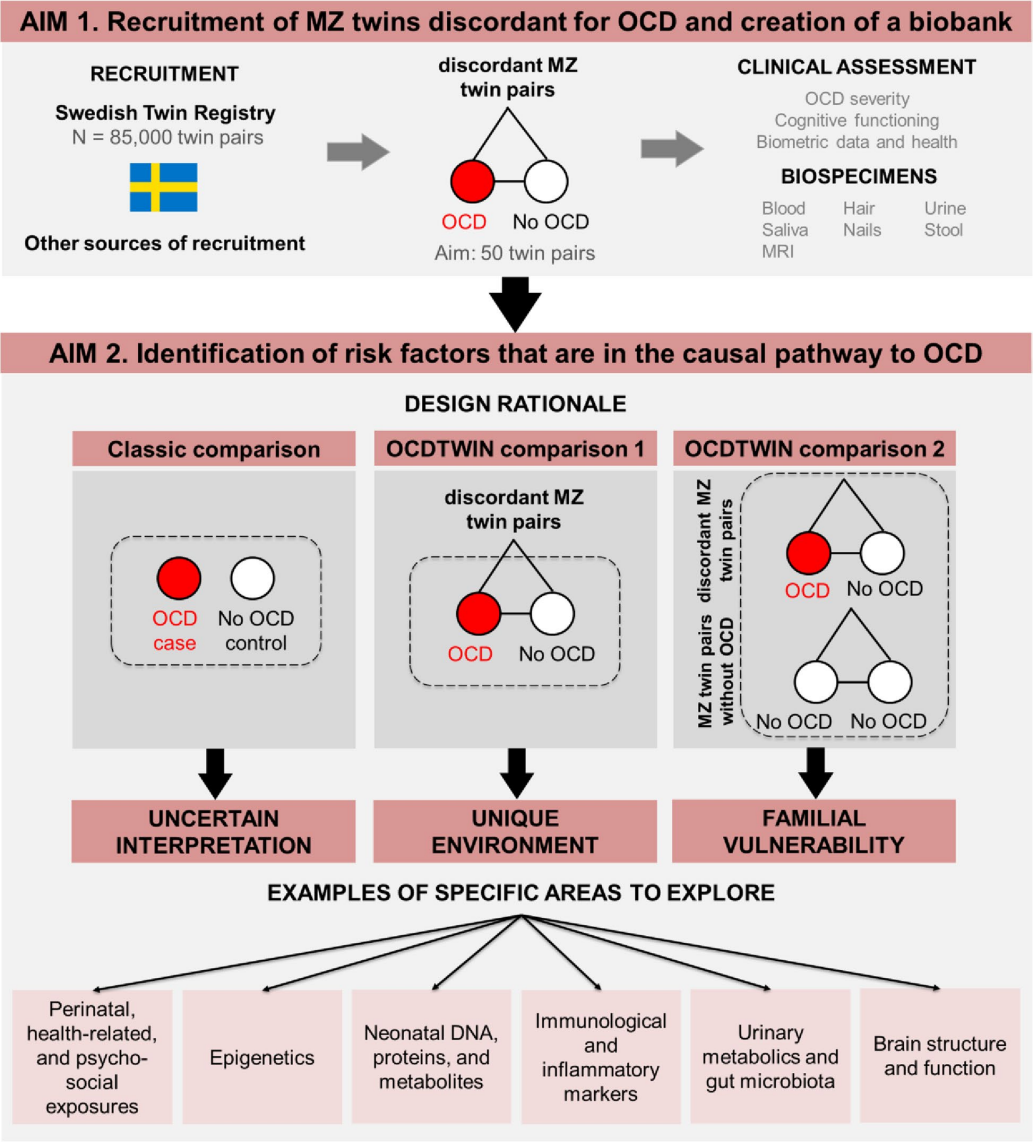
Study aims and design rationale. ODCTWIN primarily aims to recruit a cohort of MZ twins who are discordant for OCD (*Aim 1*) and identify environmental risk factors that are in the causal pathway to OCD (*Aim 2*). The results of traditional case-control designs are difficult to interpret because they are unable to effectively control for familial confounders (*Aim 2*, classic comparison). MZ twins discordant for OCD provide a unique opportunity to isolate environmental risk factors that are unique to each individual, while controlling for measured and unmeasured confounders, including shared genetic factors, and early life environmental effects (*Aim 2*, comparison 1). Even though our main interest is to identify unique environmental effects, we will also compare affected twin pairs (where at least one twin has OCD) with unaffected twin pairs to identify effects that can be attributed, at least in part, to familial vulnerability (*Aim 2*, comparison 2)

The broad study aims are two-fold (for a visual summary, see Fig. 1). In *Aim 1*, we are recruiting MZ twin pairs from across Sweden, conducting thorough clinical assessments, and building a biobank of specimens, including blood, saliva, urine, stool, hair, nails, and multimodal brain imaging. A wealth of information regarding early life exposures (e.g., perinatal variables, health-related information, psychosocial stressors) is available through linkage with the nationwide registers [6] and the Swedish Twin Registry [7, 8]. Blood spots stored in the Swedish phenylketonuria (PKU) biobank are available to extract DNA, proteins, and metabolites, providing an invaluable source of biomaterial taken at birth [9]. In *Aim 2*, we will identify variables that are in the causal pathway to OCD through within-pair comparisons of discordant MZ twins. This will allow us to isolate unique environmental factors while strictly controlling for genetic and shared environmental influences. The ultimate goal is to generate new insights into the potentially modifiable causes of OCD.

## Methods

### Aim 1. Recruitment of MZ twins discordant for OCD and creation of a biobank

#### Participants and recruitment sources

OCDTWIN aims to recruit a minimum of 50 MZ twin pairs (*n* = 100 unique individuals) discordant for OCD aged 16 years and older (see Power Considerations section below). Control twin pairs without a diagnosis of OCD are available from the ongoing Roots of Autism and Attention-Deficit/Hyperactivity Disorder (ADHD) Twin Study in Sweden (RATSS) project [10], which uses largely identical procedures. We will also recruit twin MZ pairs concordant for OCD diagnosis. By recruiting both discordant and concordant twin pairs, we will be able to appropriately represent the source population with regards to exposure variables and covariate distributions, and account for this in statistical analyses, both in within-pair analyses and standard (between individual) analyses (see General statistical framework section for details).

The twins are recruited from different sources. The main source of recruitment is the Swedish Twin Registry, the largest and most comprehensive twin register in the world [7, 11]. All the cohorts listed in Table 1 contain validated measures of obsessive-compulsive symptoms, allowing identification of MZ pairs potentially concordant or discordant for these symptoms. Furthermore, the Swedish Twin Registry has been linked with the National Patient Register [12], allowing for the identification of twins who have been diagnosed with OCD in specialist services across Sweden. Crucially, participants in the Swedish Twin Registry have provided informed consent to be contacted for research purposes. Other sources of recruitment are the Swedish OCD interest organization (*Svenska OCD-förbundet*) and media advertisements.

**Table 1:** Cohorts in the Swedish Twin Registry used for recruitment purposes and criteria for potential participant selection

**Twin cohort**	**Num. of individual twins as of November 2022**	**Obsessive-compulsive symptom measurement**	**Criteria for selection for potential inclusion**
**CATSS-18** (born 1992-)	15,400	Brief Obsessive-Compulsive Scale (BOCS) [13], *17 items*	Top 5% scores on the BOCS
**CATSS-24** (born 1992-)	7,639	Obsessive-Compulsive Inventory – Revised (OCI-R) [14], *12 items (hoarding and neutralizing scales not included)* Self-reported question: *Have you ever been diagnosed with (or suspected that you have) OCD?*	Top 5% scores on the OCI-R or answered yes to the self-reported question
**DOGSS** (born 1993–1995)	450	Kiddie - Schedule for Affective Disorders and Schizophrenia for school-aged children, Present and Lifetime Version (K-SADS-PL) [15], *obsessive-compulsive disorder categorical diagnosis*	Met diagnostic criteria for obsessive-compulsive disorder on the K-SADS-PL
**YATSS** (born 1985–1992)	6,870	OCI-R [14], *12 items (hoarding and neutralizing scales not included)*	Top 5% scores on the OCI-R
**STAGE** (born 1959–1985)	43,000	7-item obsessive-compulsive disorder questionnaire [4] Self-reported question: *Have you ever been diagnosed as having an obsessive compulsive disorder?* (yes/no)	Top 5% scores on the 7-item obsessive-compulsive disorder questionnaire or answered yes to the self-reported question

*Abbreviations*: *CATSS* Child and Adolescent Twin Study in Sweden, *DOGSS* Developmental Outcomes in a Genetic Twin Study in Sweden, *YATSS* Young Adult Twins in Sweden Study, *STAGE* Screening Twin Adults: Genes and Environment study

#### Procedures

Potential participants identified via any of the sources described above receive a study invitation letter including information about the OCDTWIN project via regular mail. Interested individuals contact the research team. This is followed by a screening phone call to assess eligibility for participation.

Inclusion criteria are: MZ twins, at least one member of the twin pair has a lifetime diagnosis of OCD, both twins consent to participate, are literate in Swedish, and are willing to travel to Stockholm for the assessment. Twins who want to participate in the study but do not wish to travel can still participate in the clinical assessments via telephone and send a subset of biological samples in the post. Information on zygosity is available from the Swedish Twin Registry, but is confirmed by genotyping of saliva or whole-blood derived DNA using a whole-genome covering SNP array [16].

Exclusion criteria include: organic brain disorder, brain injury, epilepsy or an acute mental disorder that may interfere with the evaluation (e.g., psychosis, bipolar disorder). Additional exclusion criteria for the Magnetic Resonance Imaging (MRI) scans include: previous brain surgery, metal implants or medical devices containing metal (e.g., pacemaker), claustrophobia, pregnancy, morbid obesity or large tattoos. Twins not currently eligible for the MRI scan can still participate in the remaining assessments and can have the scan at a later stage (e.g., in the case of pregnancy).

Twin pairs meeting inclusion criteria are mailed a questionnaire package (including the measures marked as “self-reported questionnaire” in Table 2) and invited for a full testing day in Stockholm. Data acquisition consists of a full day of evaluations, including a detailed clinical interview, neurocognitive testing, physical examination, collection of biological samples, and an MRI scan. Table 2 summarizes the collected information and the instruments used. All biological specimens are deposited at the Karolinska Institutet biobank, according to standard protocols.

**Table 2:** Study variables and collection

**STUDY VARIABLE**	**MEASURED BY**
**Socio-demographic and clinical information**	
Demographics	Self-reported questionnaire
Zygosity	Self-reported questionnaire, confirmed by genotyping of saliva or whole-blood derived DNA using a whole-genome covering SNP array [17]
Treatment history (medication, therapy)	Clinician-administered interview
**Psychiatric disorders**	
Structured Clinical Interview for DSM-5 (SCID-5) [18]	Clinician-administered interview
**OCD severity and symptom dimensions**	
Yale-Brown Obsessive Compulsive Scale (Y-BOCS), Symptom Severity and Checklist [19]	Clinician-administered interview
Obsessive Compulsive Inventory-Revised (OCI-R) [14]	Self-reported questionnaire
**Cognitive function**	
Similarities, Vocabulary, Information, Block design, Matrix reasoning, and Visual puzzles from the Wechsler Adult Intelligence Scale-fourth edition (WAIS-IV) [20]	Clinician-administered test
Reading the mind in the eyes (RTMITE) [21]	Clinician-administered test
Fragmented Picture Test (FPT) [22]	Clinician-administered test
Edinburgh Handedness Inventory modified [23]	Self-reported questionnaire
**Biometric data and general health**	
Height	Physical exam by nurse
Weight	Physical exam by nurse
BMI	Physical exam by nurse
Head circumference	Physical exam by nurse
Blood pressure	Physical exam by nurse
Pulse	Physical exam by nurse
Medical and Clinical Genetic Questionnaire [10]	Self-reported questionnaire
**Biospecimens**	
Blood	Collected by nurse
Saliva	Collected by nurse
Hair	Collected by nurse
Nails	Collected by nurse
Urine	Collected by participant
Stool	Collected by participant
**Brain imaging**	
High resolution structural scan (T1)	Study coordinator
Diffusion Tensor Imaging (DTI)	Study coordinator
Resting state functional Magnetic Resonance Imaging (rsfMRI)	Study coordinator
**Other measures**	
Adult Self Report (ASR) [24]	Self-reported questionnaire
Adult Autism Spectrum Quotient (AQ) [25]	Self-reported questionnaire
Sensory Profile Adolescent/Adult (SP-A) [26]	Self-reported questionnaire
Swedish Eating Assessment for Autism Spectrum Disorders (SWEAA) [27]	Self-reported questionnaire
Attention-Deficit/Hyperactivity Disorder Self Report Scale (ASRS) [28]	Self-reported questionnaire
Camouflaging Autistic Traits Questionnaire (CAT-Q) [29]	Self-reported questionnaire

Participants are reimbursed for their lost working hours. Additionally, participants receive a gift card worth SEK 500 (~ 45 €) for each of the three following parts of the study: (1) clinical assessment, (2) biological samples, and (3) MRI scan (i.e., SEK 1500 [~ 135 €] in total). For twins hailing from outside Stockholm, ground transportation or airfares and accommodation are provided.

### Aim 2. Identification of risk factors that are in the causal pathway to OCD

#### Design rationale

Genetically-informative studies, in particular those employing the discordant MZ twin design, are ideally suited to test whether the association between an environmental measure (e.g., medical complications at birth) and an observed phenotype (e.g., OCD) is likely to be consistent with a causal effect because they provide excellent control of many potential confounders, including genetic factors and shared environmental influences. Because MZ twins are genetically identical, and grow up largely in the same environment, any observed phenotypic differences between members of a MZ twin pair (e.g., one twin is affected and the other is not) may be attributable to non-shared environment. In contrast to studies comparing a sample of cases vs. controls (classic comparison, Fig. 1), or even relatives of cases, MZ twins discordant for OCD provide a unique opportunity to isolate environmental risk factors that are unique to each individual, while controlling for a myriad of measured and unmeasured confounders, such as genetic factors, sex, age, parental effects, as well as shared *in utero* and early life environmental effects (comparison 1, Fig. 1).

#### General statistical framework

Within-pair differences between affected MZ twins with OCD and their co-twins will be analyzed in a generalized estimating equation (GEE) framework, accounting for dependencies between twins in pairs using cluster-robust standard errors. In what is commonly referred to as co-twin control design, we will examine within-pair associations by analyzing data conditioned on pairs (fixed-effects regression) [30–32]. Results from these analyses are automatically adjusted for any confounding factors that are shared between twins in a pair [33], particularly genetic factors, since MZ twins are genetically identical (comparison 1, Fig. 1). Even though our main interest is to identify unique environmental effects, we will also compute a standard association (that is, individuals with OCD vs. controls, regardless of co-twin OCD status) by re-weighting the data by sampling probability and will thus recover the association in the source population, making it possible to identify effects potentially attributable, at least in part, to familial vulnerability (comparison 2, Fig. 1). In addition, all twin pairs, regardless if recruited as concordant or discordant, will contribute to analyses of within-pair associations between other variables of interest than OCD diagnosis, where they may be discordant, such as scores on OCD severity scales.

#### Register-based data

The Swedish national registers contain administrative records from the entire population prospectively collected over several decades [6]. Data from different registers can be linked by using the personal identification number assigned to all Swedish residents at birth or immigration [34]. We will have access to a wide range of early life exposures, such as perinatal and early-life health-related variables, that may have resulted in differentially exposed twins. For twins recruited via the Swedish Twin Registry [7, 8], a wealth of prospectively collected data (parent and twin-reported) on environmental exposures are available for analysis. The Child and Adolescent Twin Study in Sweden (CATSS) cohort of the Swedish Twin Registry, where most participants are recruited from, has been described in detail elsewhere [17]. Importantly, the information from the Swedish Twin Registry can be linked to the above-mentioned national registers. For a list of linked registers and examples of available variables, see Table 3. Because we carefully record the date of OCD symptom onset and diagnosis of the affected twins, we will be able to identify exposures that preceded symptom onset.

**Table 3:** Registers and examples of variables available from each participant

Register name	Examples of available variables
**Swedish national registers**	
National Patient Register [12]	Information on all inpatient and outpatient contacts at all hospitals and specialist centers. Includes primary and supplementary diagnoses based on the International Classification of Diseases codes, including somatic (e.g., autoimmune diseases, infections, allergies, respiratory diseases) and psychiatric disorders (e.g., trauma and stress related disorders, mood disorders, substance use disorders).
Medical Birth Register [35]	Information on antenatal, obstetric, and neonatal care, including mother’s parity, mother’s age, type of delivery, obstetric complications (e.g., preeclampsia, gestational diabetes, antepartum or postpartum hemorrhage), gestational age, birth weight, birth order, APGAR scores, neonatal hypoglycemia, neonatal jaundice, neonatal infections, neonatal respiratory distress, congenital malformations, etc.
Prescription Drug Register [36]	Individual-level data for all prescriptions dispensed for in- and outpatients, including type of medication registered using Anatomical Therapeutic Chemical (ATC) Classification System codes, dosage, prescription date, prescriber, pharmacy, etc.
**Swedish Twin Registry**	
	Variables collected via questionnaires in different waves, including somatic and mental health, personality development, vaccinations, substance use, physical activity or psychosocial adaptation and environment (e.g., traumatic events, school problems, friendships, bullying victimization/perpetration).

#### Epigenetics – methylation analyses

Current neurobiological models of OCD implicate epigenetic mechanisms in the etiology of OCD [37]. However, the literature is limited. As the genomes of MZ twins are identical, our design is ideally suited for the identification of epigenetic changes, potentially allowing for the observation of changes in the epigenome in absence of genetic variation between twins. DNA methylation analysis, which has been previously studied in neurodevelopmental disorders [38] and in OCD [39], will be used to determine differential methylation in the affected twin sibling, compared to the unaffected co-twin. Genome-wide methylation analysis will be used first, given the limited evidence of methylation changes in OCD. Second, we will follow the general approach of a previous epigenetic study in OCD [39], which specifically examined DNA methylation profiles of selected loci that had been associated with OCD in previous genome-wide association studies (GWAS) [40–42]. However, previous GWAS of OCD were severely underpowered. Our proposed analyses are timely as the largest GWAS study conducted to date, including approximately 45,000 cases and 30 genome-wide-significant loci, is nearing completion. We hypothesize that affected twins will exhibit differential methylation at genes identified by this GWAS, compared to their unaffected co-twins. Analyses of genome-wide methylation and methylation profiles of selected genes will be performed using array-based specific DNA methylation analysis. This array targets > 935,000 CpG sites at single nucleotide resolution, including 99% of RefSeq genes and 96% of CpG islands can be analyzed. Possibly differentially methylated regions will be confirmed by pyrosequencing or nanopore sequencing. As the blood-derived DNA is a mixture of the blood cell type specific methylation patterns, we will collect information about the cell counts as well as correct bioinformatically if there are any putative differences due to cell populations [43].

As it is a priority of OCDTWIN to identify the epigenetic effects of unique environment while controlling genetic effects, several additional genetic mechanisms will be studied in order to confirm identical genomes in affected and unaffected twins. This includes chromosomal mosaicism, post-zygotic mutation, and mutations of mitochondrial DNA [44]. To assess the landscape of genetic variants among the twins both for somatic and germline, we will use whole genome sequencing (WGS) [45] and high-density DNA microarrays. DNA microarrays can be used for detection of large CNVs using multiple analysis programs, and the variations found in samples will be compared to control twins, other available controls, and databases to identify the frequency and functionality of the variants identified. Furthermore, polygenic risk scores can be calculated and incorporated to all analyses within the OCDTWIN project. WGS can identify rare post-zygotic somatic mutations in the twins. Additionally, rare, damaging variants will be investigated for putative liability variants. Identified somatic and selected damaging germline variants will be subject to technological validation by Sanger sequencing or using digital droplet PCR.

#### Neonatal blood spots

The Swedish PKU biobank [9] contains neonatal blood spots from all children born in Sweden since 1975. Participating twins consent to the use of these blood spots to extract DNA, proteins, and metabolites, providing an invaluable source of biomaterial taken at birth. In other disorders, important discoveries have been made using neonatal blood spots. For example, persons who develop psychosis have lower levels of certain acute phase proteins (APPs) at the time of birth [46]. APPs are central to innate immune function as well as central nervous system development. Prior studies [47] have demonstrated a high genotyping call rate using whole genome amplified DNA from Swedish blood spots collected from 1975 to 2002. Two 3 mm punches from the blood spots are incubated in 200 µl 1x phosphate buffered saline for 2 h at room temperature on a rotary shaker (900 rpm), yielding an eluate of proteins such as acute phase proteins and antibodies as well as other metabolites (e.g., vitamin D, cytokines, etc.). DNA is then extracted (~ 40–150 ng), only a portion of which (10 ng) is whole genome amplified (Repli-g screening kit, Qiagen). The unamplified DNA retains methylation marks and can be used for epigenetic profiling and/or CNV validation. The amplified DNA can be used for array genotyping, exome sequencing or whole genome sequencing. These analyses will be conducted in collaboration with colleagues at the Statens Serum Institut in Copenhagen, Denmark.

#### Immunology/inflammation

Pediatric Autoimmune Neuropsychiatric Disorder Associated with Streptococcal Infection (PANDAS) can be viewed as an example of a gene-environment interaction leading to OCD [48]. In PANDAS, a relatively common infection appears to represent an environmental stressor that can trigger OCD in a few genetically vulnerable cases. In support of this idea, we have recently reported that while *in utero* and early life infections are associated with a subsequent risk of OCD, the associations attenuated to the null in sibling models [49]. This suggests that familial or genetic factors explain the association between these early-life infections and OCD. In other words, infections may only trigger obsessive-compulsive symptoms in genetically vulnerable individuals. Through register linkage, we will be able to test whether affected twins are more likely to have had documented infections in early childhood, compared to their unaffected co-twins, in OCD-discordant pairs. In addition, the following markers will be tested in blood: complete blood count (CBC), erythrocyte sedimentation rate (ESR), CRP, TSH, T4, anti-TPO, ferritin, autoantibodies (e.g., transglutaminase-Abs, ANA, Histone-Abs), creatinine, cystatin-C, ALAT, protein fractions, complements, IL-1-β, IL-6, IL-8, IL-10, and TNF-α. In line with our statistical approach, differences between affected and unaffected members of a twin pair will be attributable to disease-state (e.g., response to a chronic illness), whereas differences between affected pairs and healthy control pairs may be interpreted as being potentially attributable to trait immunological or vulnerability factors.

#### Urinary metabolics and gut microbiota

By comparing urinary metabolics and gut microbiota within and between twin pairs, we aim to explore an additional etiological pathway that has been recently suggested [50]. Using urinary samples, metabolic phenotyping will involve high-resolution proton nuclear magnetic resonance (hydrogen-1 nuclear magnetic resonance; 1 H NMR) spectroscopy coupled with mathematical modeling approaches to identify metabolic variation associated with OCD discordance in urine and plasma. Metabolic profiles are measured on a 600 MHz 1 H NMR spectrometer using standard one-dimensional NMR experiments optimized for quality, sensitivity, and solvent suppression. Liquid chromatography-mass spectrometry (LCMS) may be applied to extend the metabolic characterization of this sample set. LCMS is a complementary technique to 1 H NMR spectroscopy with greater sensitivity and wider metabolome coverage. Using fecal samples, gut microbiota will be investigated, which has emerged as an important functional node within the gut-brain axis [51]. There is increasing interest in the relative potential of the gut microbiota and allied gastrointestinal systems to modulate behavioral functions implicated in psychiatric disorders, including OCD [50]. The determination of gut microbiota will be based on the quantification of evolutionary conserved DNA sequences [52]. In microbes, ribosomal RNA (rRNA) genes are transcribed from the ribosomal operon as 30 S rRNA precursor molecules and then cleaved by RNaseIII into 16 S, 23 S, and 5 S rRNA molecules. Because 16 S rRNA is the most conserved of these three rRNAs, it is often referred to as the “evolutionary clock” and, following amplification into 16 S rDNA, is highly suitable for the identification and classification of the entire microbial community present in an environmental entity, such as the gut. The total microbial population in human fecal samples will be determined using two state-of-the-art methods, namely 16 S rDNA pyrosequencing and 16 S rDNA sequencing.

#### Brain

Individuals with OCD display subtle difficulties in neuropsychological tasks of motor and cognitive inhibition, performance monitoring, cognitive flexibility, and emotional processing [53]. Consistently, structural and functional neuroimaging studies have found involvement of specific fronto-striato-thalamic and parietal systems in OCD [53], although causal relationships cannot be established. It is unclear whether differences between OCD cases and controls represent pre-existing vulnerabilities that precede the onset of the disorder or are environmentally or behaviorally mediated. In support of the former view, a number of studies have found that individuals with OCD and their unaffected first-degree relatives share similar cognitive and neural features (e.g., [54–56]). However, as siblings only share about 50% of their genes, it is still unclear whether these findings reflect genetic vulnerability or environmentally-mediated risk factors. In support of the latter view, variables such as living with a chronic illness are suspected to induce neuroplastic changes in the brain of individuals with OCD [57–60]. Similarly, medication may represent another unique environmental event affecting brain structure in OCD, as indicated by recent mega-analyses [61, 62]. The discordant MZ design is ideally suited to understand what brain findings may be secondary to environmental exposures, such as use of medication.

MRI data are acquired on a 3T General Electric 750 MR scanner (equipped with a 32-channel head coil) at the MR Research Center at Karolinska Institutet. T1-weighted images are acquired using a high-resolution BRAVO 3D sequence, using the following parameters: TR/TE = 8.2/3.2; 172 slices; FOV: 240; 256 × 256; 1 × 0.94 × 0.94 mm; flip angle = 12 degrees. Voxel-based morphometry analyses will determine whether gray matter volume differences in cingulate cortex and basal ganglia areas observed in previous meta-analyses [63] can be attributed to unique environmental risk.

Diffusion tensor imaging (DTI) measurements of white matter microstructure are acquired using High AngulaR Diffusion Imaging (HARDI) with 60 directions and 61 slices, Dual spin Echo Epi2ks axial; TR/TE: 8000/99; FOV 96 × 96; 8 b0 images, b-value: 1000 s/mm^2^. Fractional anisotropy (FA) and white matter volume analyses will help determine whether white matter differences observed in previous meta-analyses [64] and mega-analyses [65] are associated with unique environmental risk factors.

Resting-state functional magnetic resonance imaging involves acquisition of 205 echo-planar images (EPI) using the following parameters: 45 slices; interleaved ascending slice order; TR/TE = 3000/30ms; FOV = 288; 96 × 96; 3 × 3 × 3 mm; flip angle = 90 degrees. Degree connectivity analysis will be performed [66], revealing local and distant “hubs” of connectivity, as well as traditional seed-based whole-brain correlation analysis to determine striatal connectivity [67, 68] and default mode network (DMN) [69].

Spatial associations between within-pair differences in whole-brain measures and whole-brain gene expression patterns will be explored. The Allen Human Brain Atlas [70] will be used to test for associations between brain structure and connectivity differences (results from within-pair comparisons) and gene expression in a previously described manner [71–73] without requiring information from blood or saliva samples, which can however potentially be integrated in subsequent analyses [73]. The spatial similarity between transcriptional profiles of the entire transcriptome atlas and within-pair differences in brain measures observed in our study population will be quantified. Histogram distributions of spatial similarity values will reveal genes where the genetic expression pattern is significantly associated with brain structure and connectivity maps. Moreover, we will particularly focus on neurogenetic processes by investigating specific gene ontology (GO) term analysis for “neuro” annotations, as described previously [71, 72]. Finally, we will focus on specific genes strongly suspected to be associated with OCD (such as DLGAP3 [74–77] or NRXN1 [78]) and also new genes uncovered in the latest GWAS.

#### Power considerations

**Fig. 2:**
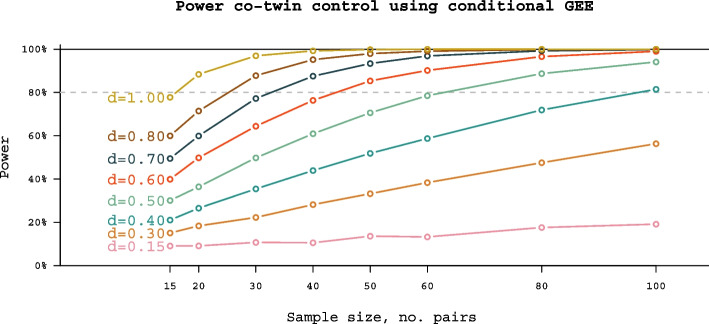
Number of discordant twin pairs needed for power per different effect sizes. “*d*” refers to average difference, measured in standard deviations, between affected and unaffected twins, similar to Cohen’s *d*

Given the novelty of the approaches presented, power analyses are necessarily tentative. Although we have a variation in distribution of variables, we have performed a power calculation for continuous, normally distributed variables in a co-twin control design using GEE analytic framework (i.e., fixed-effects linear regression) (see Fig. 2). With 50 discordant MZ pairs, we will have approximately 80% power to detect medium to strong associations (Cohen’s *d* of approximately 0.6). Publications emerging from the ongoing RATSS project [79, 80] suggest that the proposed sample sizes can yield meaningful results.

## Discussion

To our knowledge, OCDTWIN represents the world’s only cohort of MZ twins discordant or concordant for OCD. The project hopes to generate unique insights into environmental risk factors that are in the causal pathway to OCD, some of which have the potential of being actionable targets. If successful, this could be a first step towards fulfilling the long-held ambition of preventing the development of OCD or, if this were not possible, intervene as early as possible to prevent the long-term medical [81] and socio-economic [82, 83] consequences of the disorder.

Some challenges for the success of the project are participant recruitment, uncertainty regarding statistical power for some of the proposed analyses, and interpretation of the results. Initially, we aim to recruit at least 50 discordant pairs of twins. At the time of writing (May 2023), a total of 43 MZ twin pairs (86 individuals) have already been recruited. Twenty-one of those MZ pairs are discordant for OCD diagnosis and 22 are concordant. Control twins are available from the parallel RATSS study [10]. Our main recruitment source, the CATSS cohort within the Swedish Twin Registry [17], is still actively recruiting at a rate of approximately 3,000 new twins per year, providing a sustained source of potential study participants. Data collection will continue for at least the next two years. If we secure additional funds, we aim to continue recruiting participants beyond the planned 50 pairs, thus increasing statistical power. The study is currently limited to Swedish residents and to participants who are 16 years or older. However, we may consider expanding to twin pairs from other countries in the future. There is a risk that some of the younger twins identified as unaffected have not had time to develop OCD by the time of their participation, as the youngest participants may be 16 years old. We plan to follow up the twins in the registers to capture any new diagnoses of OCD after they have been recruited to OCDTWIN. Some of the described methods and analysis plans may be obsolete by the time we are ready for data analysis. We are collecting hair and nails but have no specific plans for analysis at the time of writing. We will closely follow methodological developments in the field.

While the primary aim of OCDTWIN is the identification of environmental risk factors that are in the causal pathway to OCD, we will collect a wide range of exposures from birth (e.g., perinatal complications, birth order, birth weight), childhood (e.g., early infections, bullying and other traumatic experiences), and up to the time of participation in the study (e.g., current medication use). While the interpretation of results regarding early exposures will be relatively straightforward because these exposures will precede the onset of OCD symptoms, the interpretation of results based on more recent exposures will be more challenging. For example, differences between affected and unaffected twins on a given brain measure could be attributable to environmental exposures accumulated during a lifetime, including changes secondary to chronic illness or medication use. Even in this scenario, the results will still be informative because the nature of the design minimizes the influence of genetic and shared environmental factors, and an association could reveal important, potentially actionable mediators. However, the interpretation of the results will differ according to each specific exposure and whether temporal precedence can be clearly established.

There are additional challenges associated with the discordant MZ twin design. Our approach assumes that MZ twins are genetically identical. However, post-zygotic mutations are known to occur and can be specific to one twin in a pair [45], which could explain OCD discordance in some pairs. On the other hand, this will provide a unique opportunity for genetic discovery. Another potential challenge is twin chorionicity, which is often unknown for adult twins. MZ twins can be sub-classified according to whether they shared the same placenta or not. For example, in a schizophrenia study, concordant MZ pairs were estimated to be more likely to have shared a single placenta, whereas discordant MZ pairs appeared more likely to have separate placentas [84]. Whether and how post-zygotic mutations and chorionicity can impact the interpretation of our results is unclear but will be considered.

The project is expected to generate many scientific outputs. All resulting papers will be deposited in preprint repositories (e.g., bioRxiv, PsyArXiv) to ensure immediate access to the scientific community. We will publish the results in specialized peer-reviewed journals that allow open access formats. Through partnership with other researchers who are collecting similar twin data in other disorders in Sweden, it may be possible to establish which findings are specific to OCD or shared with other neuropsychiatric conditions. OCDTWIN will collect nearly the same data as the RATSS study [10], which focuses on autism and ADHD. Similarly, the ongoing CREAT (Comprehensive Risk Evaluation for Anorexia nervosa in Twins) study focuses on MZ twins who are discordant for anorexia nervosa [85]. Both these cohorts will provide additional opportunities for collaboration.

## Data Availability

Due to European legislation, the data will not be deposited in public repositories. However, the data can be made available to the international research community for formal collaborations upon reasonable request and adequate data transfer agreements that comply with Swedish and European law. Collaboration and data requests should be addressed to the corresponding author (david.mataix.cols@ki.se).

## Abbreviations

3T: 3 Tesla
ADHD: Attention-Deficit/Hyperactivity Disorder
ALAT: Alanine aminotransferase
ANA: Antinuclear Antibody
Anti-TPO: Thyroid Peroxidase antibodies
APGAR: Appearance, Pulse, Grimace, Activity, Respiration
APP: Acute Phase Protein
AQ: Adult Autism Spectrum Quotient
ASR: Adult Self Report
ASRS: Attention-Deficit/Hyperactivity Disorder Self-Report Scale
ATC: Anatomical Therapeutic Chemical
BMI: Body-Mass Index
BOCS: Brief Obsessive-Compulsive Scale
BRAVO 3D: Axial MRI 3D Brain Volume
CAT-Q: Camouflaging Autistic Traits Questionnaire
CATSS: Child and Adolescent Twin Study in Sweden
CBC: Complete Blood Count
CNV: Copy Number Variation
CpG: Cytosine-phosphate-Guanine
CREAT: Comprehensive Risk Evaluation for Anorexia nervosa in Twins
CRP: C-Reactive Protein
DLGAP3: DLG Associated Protein 3
DMN: Default Mode Network
DNA: Deoxyribonucleic Acid
DOGSS: Developmental Outcomes in a Genetics twin Study in Sweden
DSM-5: Diagnostic and Statistical Manual of mental disorders, fifth edition
DTI: Diffusion Tensor Imaging
EPI: Echo-Planar Images
ESR: Erythrocyte Sedimentation Rate
FA: Fractional Anisotropy
FOV: Field Of View
FPT: Fragmented Picture Test
GEE: Generalized Estimating Equation
GO: Gene Ontology
GWAS: Genome-wide Association Study
HARDI: High AngulaR Diffusion Imaging
IL: Interleukin
K-SADS-PL: Kiddie-Schedule for Affective Disorders and Schizophrenia for school-aged children, present and lifetime Version
LCMS: Liquid Chromatography-Mass Spectrometry
MHz: Megahertz
MRI: Magnetic Resonance Imaging
MZ: MonoZygotic
NMR: Nuclear Magnetic Resonance
NRXN1: Neurexin 1
PANDAS: Pediatric Autoimmune Neuropsychiatric Disorder Associated with Streptococcal Infection
PCR: Polymerase Chain Reaction
OCD: Obsessive-Compulsive Disorder
OCI-R: Obsessive-Compulsive Inventory-Revised
PKU: PhenylKetonUria
RATSS: Roots of Autism and Attention-Deficit/Hyperactivity Disorder Twin Study in Sweden
rDNA: ribosomal Deoxyribonucleic Acid
RefSeq: Reference Sequence
rsfMRI: resting state functional Magnetic Resonance Imaging
rRNA: ribosomal Ribonucleic Acid
RTMITE: Reading the mind in the eyes
SCID-5: Structured Clinical Interview for DSM-5 Axis I disorders
SEK: Swedish Krona
SNP: Single Nucleotide Polymorphism
SP-A: Sensory Profile-Adolescent/Adult
STAGE: Screening Twin Adults:Genes and Environment study
SWEAA: Swedish Eating Assessment for Autism spectrum disorders
T4: Thyroxine
TNF-α: Tumor Necrosis Factor-α human
TR/TE: Repetition Time/Echo Time
TSH: Thyroid Stimulating Hormone
WAIS-IV: Wechsler Adult Intelligence Scale-IV
WGS: Whole Genome Sequencing
YATSS: Young Adult Twins in Sweden Study
Y-BOCS: Yale-Brown Obsessive-Compulsive Scale
